# Prediction of amyloid and tau status in nondemented older adults using tree‐based ensemble models

**DOI:** 10.1002/alz.090593

**Published:** 2025-01-09

**Authors:** Younghoon Seo, Hwamee Oh

**Affiliations:** ^1^ Bowdoin College, Brunswick USA; ^2^ Brown University, Providence, RI USA

## Abstract

**Background:**

Predicting amyloid and tau status in nondemented older adults with AD pathologies using more affordable and accessible measures can facilitate clinical trials by reducing the screen failure rate. The goal of the present study was to develop tree‐based ensemble models to predict PET‐based amyloid and tau burden using non‐invasive measures.

**Method:**

Two datasets, amyloid (Aβ; n = 1062) and tau (n = 410), from the Alzheimer’s Disease Neuroimaging Initiative (ADNI) database were used to predict the biomarker load in the subjects with normal cognition and mild cognitive impairment. Amyloid PET with the [18F]Florbetapir tracer was used as the gold‐standard measure for binary amyloid status classification), while tau PET with the [18F]Flortaucipir tracer was used for the three‐stage (low, intermediate, and high) determination. We trained random forest (RF), extreme gradient boosting machine (XGBoost), and light gradient boosting machine (lightGBM) models using different combinations of demographic, neuropsychological, APOE genotype, and volumetric MRI data, and measured the model performance using area under the receiver operating curve (AUROC).

**Result:**

The performance of baseline model with demographics showed modest performance for Aβ (RF = 0.665, XGB = 0.650, LGBM = 0.659). Subsequent additions of features improved the predictive performance, with the model using demographic data, cognitive data, and volumetric MRI measures demonstrating the highest performance (RF = 0.762, XGB = 0.763, LGBM = 0.761). Meanwhile, the baseline model achieved modest performance for the three‐stage tau classification (RF = 0.643, XGB = 0.654, LGBM = 0.643), and the further addition of features improved the performance, with the feature combination of demographic data, cognitive, volumetric MRI measures, and continuous Aβ PET SUVRs achieving very good performance (RF = 0.799, XGB = 0.801, LGBM = 0.800). SHAP summary plots showed that age, entorhinal cortex volume, and neuropsychological and functional measures were important for Aβ classification, while Aβ load, high global cognition scores, hippocampal and middle temporal gyrus volume were shown to predict tau status.

**Conclusion:**

Without using amyloid and tau PET, tree‐based ensemble machine learning models predict amyloid and tau status among nondemented older adults with modest to very good performance and could be incorporated for future clinical trials.

